# Users’ Modifications to Electronic Nicotine Delivery Systems: Content Analysis of YouTube Video Comments

**DOI:** 10.2196/38268

**Published:** 2022-08-12

**Authors:** Yachao Li, David L Ashley, Lucy Popova

**Affiliations:** 1 Department of Communication Studies The College of New Jersey Ewing, NJ United States; 2 Department of Public Health The College of New Jersey Ewing, NJ United States; 3 School of Public Health Georgia State University Atlanta, GA United States

**Keywords:** ENDS modifications, YouTube, comments, vaping, content analysis

## Abstract

**Background:**

User modifications can alter the toxicity and addictiveness of electronic nicotine delivery systems (ENDSs). YouTube has been a major platform where ENDS users obtain and share information about ENDS modifications. Past research has examined the content and characteristics of ENDS modification videos.

**Objective:**

This study aims to analyze the video comments to understand the viewers’ reactions to these videos.

**Methods:**

We identified 168 YouTube videos depicting ENDS modifications. Each video’s top 20 most liked comments were retrieved. The final sample included 2859 comments. A content analysis identified major themes of the comment content.

**Results:**

Most comments were directed to creators and interacted with others: 952/2859 (33.30%) expressed appreciation, 135/2859 (4.72%) requested more videos, 462/2859 (16.16%) asked for clarification, and 67/2859 (2.34%) inquired about product purchases. In addition, comments mentioned viewers’ experiences of ENDS modifications (430/2859, 15.04%) and tobacco use (167/2859, 5.84%); about 198/2859 (6.93%) also indicated intentions to modify ENDSs and 34/2859 (1.19%) mentioned that they were “newbies.” Moreover, comments included modification knowledge: 346/2859 (12.10%) provided additional information, 227/2859 (7.94%) mentioned newly learned knowledge, and 162/2859 (5.67%) criticized the videos. Furthermore, few comments mentioned the dangers of ENDS modifications (136/2859, 4.76%) and tobacco use (7/2859, 0.24%). Lastly, among the 15 comments explicitly mentioning regulations, 13/2859 (0.45%) were against and 2/2859 (0.07%) were supportive of regulations.

**Conclusions:**

The results indicated acceptance and popularity of ENDS modifications and suggested that the videos might motivate current and new users to alter their devices. Few comments mentioned the risks and regulations. Regulatory research and agencies should be aware of online ENDS modification information and understand its impacts on users.

## Introduction

Electronic nicotine delivery systems (ENDSs), also known as vapes, e-cigarettes, and e-hookahs, have become increasingly popular in the United States [1]. Some smokers may utilize ENDSs to quit smoking and some randomized control trials suggest that, under certain conditions, ENDSs may improve smoking cessation compared with nicotine replacement therapy [2]. However, current evidence from population studies indicates no significant association between ENDS use and increased smoking cessation among cigarette smokers [3]. Emerging research also suggests that ENDS use has both short- and long-term health risks [4-6], such as burn injuries [7], lung inflammation, and pulmonary fibrosis [8], and low birth weight associated with parental ENDS use [9]. Moreover, ENDS products often include highly modifiable features that allow users to alter device, liquid, and aerosol characteristics, which may cause even more harmful consequences [10]. Indeed, The Centers for Disease Control and Prevention (CDC) urges ENDS users not to add substances or modify the products not intended by the manufacturer [11].

ENDS modifications include product misuse and tampering unintended by the manufacturers, as well as alteration, customization, adjustment, and user choice of e-liquid or accessories made within manufacturer parameters [12,13]. For instance, some users may alter the liquid materials to be aerosolized, such as making their own e-juice, adding substances such as cannabis, or substituting manufactured liquids with materials of unknown composition and origin [12,14]. Other common practices include modifying heating coils and changing battery voltage to increase levels of nicotine delivery, produce larger clouds, and experience different throat hit [12,14]. While users consider the ability to customize nicotine levels and flavors an attractive feature of ENDS products [10], ENDS modifications can expose users to higher levels of harmful substances in the aerosol when they increase power to the coil [15,16]. Other harms related to ENDS modification include overheating and explosion-related injuries [17,18], use of illicit substances [19], and clinical nicotine toxicity [20]. Moreover, the availability of certain flavors encourages youth use [1]. Thus, ENDS modifications could change the toxicity and addictiveness of the products, inhibit cessation, and increase initiation of ENDS use.

Given the popularity of ENDS and the health risks related to modifications, more research is needed to understand users’ attitudes toward modifications and behaviors when modifying ENDS. As one of the primary video-sharing sites across the world, YouTube has been identified as a major platform where ENDS users share and obtain information about ENDS products and modifications [12,21,22]. To date, 2 studies [13,23] have examined the characteristics and content of YouTube videos depicting ENDS modifications. One study found that videos depicting unorthodox use (unintended by the manufacturer) were 3 times more prevalent than videos depicting orthodox use (intended by the manufacturer) [23]. Another study highlighted several concerning trends in ENDS modification videos, including lack of warnings, adding marijuana derivatives to e-liquids, and positive portrayal of ENDS devices and modifications [13]. While both studies provide valuable insights into how users modify ENDSs and identify features of the modification videos, we know little about viewers’ reactions to those YouTube videos, which may be mined for understanding the potential impacts of online information on people’s ENDS modification attitudes and behaviors.

One way to understand viewers’ reactions to YouTube videos is to study the comments left on the videos. The comment function allows users to directly respond to the video content and to express their opinions [24]. The data collection is also unobtrusive, providing relatively accurate valuable insights into how viewers naturally think about the videos and the issues portrayed in the videos. Thus, we conducted a content analysis of user comments on ENDS modification videos. The research objectives are to identify common themes of those comments and to explore YouTube users’ attitudes toward ENDS use and modifications. The results would complement prior research on ENDS modifications and studies of YouTube modification videos, as well as provide a better understanding of the potential effects of the videos on viewers.

## Methods

### Data Collection

We searched YouTube on March 15, 2019, to identify videos depicting ENDS modifications. A new account was created on the incognito (private) mode to minimize the impacts of browsing history on search results. A total of 28 search phrases, derived from interviews with ENDS users [12] and the literature [25], were utilized, such as “vape DIY (ie, do it yourself),” “vape e-juice custom build,” “vape dripping DIY,” and “vaping modif* custom build.” The full search terms and video identification procedures are reported elsewhere [13]. The top 10 most viewed videos for each search phrase were identified (n=280 videos). Trained coders then reviewed the videos and removed duplicates, non-English videos, and those not presenting ENDS modifications, resulting in 168 videos. The oldest video was posted on May 1, 2013, and the most recent video was posted on March 14, 2019. A video featured one or multiple types of ENDS modifications. Specifically, modifications to the coil were the most frequently portrayed in the videos (70%), followed by modification of e-liquids (26%), battery modifications (8%), and refilling nonreusable pods with e-liquids (5%) [13].

Sorted by the number of likes to the comments, each video’s top 20 comments were retrieved. Notably, only initial comments directed to the videos were retrieved. Replies to comments were rare in our data collection and excluded. If a video had fewer than 20 comments, then all comments were included. A total of 3103 comments were eventually identified. After removing comments that were non-English, the final data set included 2859 comments on ENDS modification videos. The oldest comment was posted 8 years ago, and the most recent comment was posted on March 14, 2019. Results on the content analyses of videos have been previously reported [13].

### Coding Procedures

The first author and a research assistant served as coders for this study. First, the 2 coders reviewed the comments multiple times to become familiar with the data. Next, open and axial coding [26] was conducted to identify prevalent types of comment content and create categories. As an interpretive process, open coding involves describing, naming, and classifying the observed data. Axial coding is an inductive process aimed at identifying higher-level concepts that organize subordinate types into broader categories [26]. In the open coding process, each coder independently generated a list of topics. Then, they compared the degree of overlap between their lists. During the axial coding phase, common topics were combined into overarching categories. In total, we identified 13 subordinate types of comment content, which were then grouped into 4 major categories. See Table 1 for examples and frequencies.

**Table 1:** Examples and frequencies of types of content in the video comments (n=2859).

Category/type^a^			Example		Value, n (%)^b^
**1. Interactions with creators and others**					1527 (53.41)
	1.1. Appreciation and compliment	*Thank you for showing how to change cotton.*		952 (33.30)	
	1.2. Request for more videos	*Hey man! Love your tutorials! Was wondering if you would make a video for the sx33 chip?*		135 (4.72)	
	1.3. Clarification and advice seeking	*Do you think there is a way to allow the resistance meter to be used longer than 3s?*		462 (16.16)	
	1.4. Purchase inquiry	*Where can you purchase replacement parts?*		67 (2.34)	
**2. Modification and tobacco use behaviors**					782 (27.35)
	2.1. Experiences of ENDS^c^ modifications	*Just made my own e-juice today. Working on prototyping adjustable airflow.*		430 (15.04)	
	2.2. Experiences of tobacco use	*I started smoking at 14 but switched to vaping in college to quit smoking cigarettes.*		167 (5.84)	
	2.3. Modification intentions	*The coil I ordered just arrived today. Now I’ll try it the way you suggested.*		198 (6.93)	
	2.4. New to ENDS use and modifications	*I’m brand new to vaping. This video helped me out a lot. Thanks dude.*		34 (1.19)	
**3. Modification knowledge**					727 (25.43)
	3.1. Providing additional info	*Cool! Also, if you choose to buy flavoring for E-Liquid use, they must be Diacetyl Free.*		346 (12.10)	
	3.2. Gained new knowledge or skills	*Wow! I finally ran into the right video and know how to build now.*		227 (7.94)	
	3.3. Criticism and “better” alternatives	*This is wrong. For Scottish roll, you should remove hard parts. Better flavor and wicking.*		162 (5.67)	
**4. Risks and safety**					143 (5)
	4.1. Dangers of ENDS modifications	*This causes way too much dry burning. It is ridiculously dangerous and stupid.*		136 (4.76)	
	4.2. Health risks of tobacco use	*Now that’s how you get popcorn lung.*		7 (0.24)	
**5. Regulation attitudes**					15 (0.52)
	5.1. Antiregulation	*US government is absolutely ridiculous, pretty soon they’re going to regulate our toilet paper.*		13 (0.45)	
	5.2. Neutral	N/A^d^		0 (0)	
	5.3. Proregulation	*Just forbid them (combustible products) as they’re a major health concern and can cause several types of cancer.*		2 (0.07)	

^a^Categories and types are not mutually exclusive. A comment may include multiple types of content (eg, having both Types 1.1 and 3.1).

^b^The value represents the number (percentage) of comments that included at least one content type, which may be smaller than adding up the numbers (percentages) of all comment types.

^c^ENDS: electronic nicotine delivery system.

^d^N/A: not applicable.

A codebook was developed based on the open and axial coding processes. We were also interested in whether and how viewers mentioned tobacco and ENDS modification regulations in their comments. Thus, a fifth category, “regulation attitudes,” was added to the codebook, including 3 types of content: antiregulations, neutral toward regulations, and proregulations (Table 1). The unit of analysis was each comment. We coded for the presence (1=presence, 0=absence) of each type of content. Types of content were not mutually exclusive. For instance, the comment, “I’m not new to vaping but I am new to the RDA (rebuildable dripping atomizer) and this video was very helpful and informative. Thank you for doing the video. I learned A LOT from you,” was coded as “appreciation and compliment” and “gained new knowledge or skills.” The 2 coders both coded a random 30% of the sample (n=900). Intercoder reliability was high, with Krippendorff α values ranging from .89 to .92. Disagreements were resolved through discussion. The remaining 1959 comments were divided evenly and randomly assigned to each coder.

### Data Analysis

Data were analyzed using SPSS version 27 (IBM, Inc.). We performed descriptive statistics to assess the frequency of each comment type and category.

### Ethical Considerations

Ethics approval is not needed because the study only analyzed publicly available data and the results do not contain any identifiable information.

## Results

Comments on ENDS modification YouTube videos included 5 categories: interactions with creators and others, modification and tobacco use behaviors, modification knowledge, risks and safety, and regulation attitudes. Each category comprised several subordinate types of comment content. On average, each comment included 17.35 words (SD 25.81), with a range from range 1 to 569.

### Interactions With Creators and Others

More than half (1527/2859, 53.41%) of the comments included the content directed to video creators or interactions with other audience members, in which the viewers complimented and appreciated the creators, requested more videos from the producers, asked for clarification, and inquired about product purchases. Specifically, about 1 in 3 comments (952/2859, 33.30%) thanked the video producers for creating the videos or praised the video content and attributes: “Thank you for showing how to change cotton,” “I really appreciate this well-detailed tutorial,” and “You put a lot of work in your video. Thanks.” A few (135/2859, 4.72% of comments) requested the creators to make more videos in general or about a particular product or ENDS modification. For instance, one comment read, “We’d like to see a Final Boss Vapes Review!” Others asked, “Can you do a review on the goblin mini-RTA please?” and “Can you do a video about how to change the coil and cotton?”

In addition, viewers asked questions about ENDS modifications in their comments. Some (462/2859, 16.16%) sought advice on or clarification for ENDS modifications. For instance, “(If I) wire the computer supply to 5v, do you think it will be safe?” “So if I have two 35a batteries, will they not work with the fuses?” and “So you are saying that the old coil is not recyclable/re-usable??” Others (67/2859, 2.34%) inquired what and where to purchase products for ENDS use and modifications. Examples included, “Can you give me a list of things to salvage them from or where to buy them online?” “How can I buy it?” and “Smok alien vs. vaporesso revenger, which one should I buy?” Notably, given that the comments are often visible to everyone, while the comments might initially be directed to the video creators, those questions could be reviewed and answered by both the creators and other viewers.

### Modification and Tobacco Use Behaviors

More than 1 in 4 (782/2859, 27.35%) comments mentioned viewers’ own experiences and intentions of ENDS modifications and tobacco use. This category represents how users were involved with and planned to engage in ENDS modifications. Specifically, 15.04% (430/2859) of comments described viewers’ past and current experiences of modifying ENDSs. For example, one comment stated, “I’ve just made a 4x32awg rods build then I twisted both ends to make it more of a clapped cable look that ohm’d out at .51.” Another said, “I made one last week to see how I was vaping in 213. And I can surely say that I’m much happier with my builds now, with a lot of surface area and organic cotton.” Moreover, 5.84% (167/2859) of comments mentioned viewers’ tobacco use experiences without explicitly referring to ENDS modifications. Some described their current ENDS use (eg, “I vape at 25 watts on my baby alien”), whereas others mentioned switching from smoking to vaping: “I used to smoke when I was 15, then switched to vapes,” “I switched from smoking 6 a day to vaping and I’m a thousand times healthier for it,” and “Vape is mainly for quitting cigarettes but honestly it could be enjoyed by anyone.”

Notably, 6.93% (198/2859) of comments indicated viewers’ intentions to modify ENDS devices or to change how they modified their ENDS products, especially after watching the modification videos. One comment said, “I made that coil in minutes with 24 gauge. You are right (that) it burns hot but easy to build. It was too much for my RDA. Thanks for the video! I will try it again with a better bigger RDA.” Other examples included, “Downloaded your pdf and printed it, I will try a build soon!” “I will try lowering the % of flavorings and see if that does the trick,” and “Thanks for the tutorial, lady, I am going to have fun with those.” In addition, 1.19% (34/2859) of comments explicitly indicated that the viewers were new to ENDS use or ENDS modifications: “I just got my DOVPO 5., a beginner actually starting vaping a week ago,” “I am a newbie at the vape game so thank you!” and “I am a newbie to cloud chasing.” The comments suggest that ENDS modification videos may motivate ENDS use and modifications.

### Modification Knowledge

In addition to modification behaviors, a quarter (727/2859, 25.43%) of comments addressed users’ knowledge of ENDS modifications. In these comments, viewers provided additional information to complement the video content, indicated that they gained new knowledge or skills, and criticized the video and offered “better” ways to modify ENDS. First, 12.10% (346/2859) of the comments added more tips or recommendations for ENDS modifications. One comment read, “This is probably the best way to mix especially when dealing with small batches like when developing a new flavor. The smaller the batch the more important it is to keep every measurement as accurate as possible...All new DIYers should take the time to get those gravity numbers and mix by mass instead of volume.” Another comment added to the video, “You are right, there is some basic stuff to know when vaping, and quality of CBD is important too!...I also buy on plantandhemp.com, you know them? Good brands and they only work with quality verified brands, I use them a lot!”

Viewers stated in their comments (227/2859, 7.94%) that they have learned specific knowledge and skills related to ENDS and ENDS modification after watching the videos. Examples included, “In the first 3 minutes you gave me a better understanding of voltage/p.d. than I ever had in physics class,” “Thanks so much for this. This helped explain ohms law in a very easy to understand way and helped me get started on everything I need to know,” and “This is exactly what I was looking for to fully understanding the principles of vaping.” However, 5.67% (162/2859) of comments disagreed with the videos and mentioned “better” alternatives to ENDS modifications. One comment read, “You are soooo incredibly wrong on PG (Propylene Glycol). Do a little more research prior to posting a video on such a vast platform.” Another comment stated, “Awesome. But I wouldn’t use the syringe to stir, because it’ll clog and gunk it up quicker and you’ll have to replace the syringes more often.”

### Risks and Safety

Only 5% (143/2859) of comments mentioned the health risks and safety concerns of ENDS modifications and tobacco use. Among those comments, the majority (4.76%, 136/2859, of all comments) focused on the risks of ENDS modifications. Some viewers raised their concerns: “Copper? Isn’t it toxic...Will cause cancer,” “Very cool for learning purposes but I would not recommend using that. Very dangerous elements you are using such as copper and zinc,” and “This is how you burn your [expletive] hands.” Others mentioned the actual adverse consequences of ENDS modifications: “My friend is in hospital because of this,” and “Did this step-by-step and caught on fire.” In addition, 7/2859 comments (0.24%) explicitly mentioned the negative health effects of tobacco products: “Just forbid them (combustible products) as they’re a major health concern and can cause several types of cancer,” “Vaping is too dangerous,” and “It (vaping) is as bad as smoking. It has toxic chemicals too.”

### Regulation Attitudes

We added this last category to the codebook to explore whether and how viewers mentioned tobacco and ENDS modification regulations in their comments. Only 15/2859 (0.52%) comments explicitly mentioned regulations, among which 13/2859 (0.45%) comments were against tobacco and ENDS modification regulations. One comment stated, “If the FDA bans flavors, then the US economy will sink simply because it’s keeping a LOT of people working in vape shops, e-liquid makers, marketing people who make labels, etc. It will be the worst decision the FDA has ever made.” Another comment also expressed concerns about ENDS regulations, “Vaping is becoming so popular that the FDA now doesn’t think it’s a good enough option to quit smoking and wants to ban flavored liquid other than tobacco :(.” No comments were neutral toward regulation. Only 2/2859 (0.07%) comments were supportive of regulations: “Seriously, this is probably why the FDA is fighting to cripple the vaping industry. Vaping may be a safer alternative to smoking, but it won’t stay that way for long if these mods keep getting more, and more powerful. If this keeps up, smoking may eventually become the safer alternative.”

## Discussion

### Principal Findings

Given the emerging evidence that ENDS modifications may result in adverse health consequences [10,15,16] and the popularity of YouTube to share and obtain information about ENDS products and modifications [12,21,22], this study aimed to explore how viewers respond to modification videos and discuss ENDS use and modifications. Specifically, we analyzed the common topics of users’ comments left on YouTube modification videos, most of which featured coil, e-liquid, and battery modifications. A content analysis identified 5 common categories and various subordinate types of comment content. The results suggest several concerning trends, including the positive reactions to ENDS modifications, potential motivating effects of modification videos, and lack of mentions of ENDS risks and regulations.

Our results showed that about 1 in 5 comments mentioned viewers’ own experiences of ENDS modifications and use. In addition, nearly 1 in 3 comments thanked the creators for the videos. About 5% (135/2859, 4.72%) of comments also included requests for more videos. An interview study of ENDS enthusiasts showed that while the prevalence of ENDS modifications might have peaked a few years ago, some hobbyists continued to build their own coils and batteries, and many users continued to misuse e-liquids [12]. Likewise, our results also revealed the acceptance and popularity of coil modification, battery alternation, and e-liquid customization. Moreover, in 18.50% (529/2859) of the comments, viewers inquired about product purchases, asked for clarifications, and sought advice about ENDS modifications from the video creators and other viewers. This demonstrates the demand for and appreciation of modification information.

Another concerning finding is that by providing ENDS modification information in an educational form, YouTube modification videos may motivate viewers, especially young audiences, to use ENDSs and engage in ENDS modifications. A prior content analysis revealed that most YouTube videos had positive portrayals of ENDS modifications without safety warnings [13]. Our results showed that 7.94% (227/2859) of comments explicitly mentioned that the viewers had learned new skills and knowledge related to modifications of their devices and e-liquids. Nearly 7% (198/2859, 6.93%) of the comments also indicated that the viewers intended to modify their ENDS devices or changed their modification activities after watching the videos. Thus, exposure to modification videos may result in positive attitudes toward ENDS modifications, increased modification knowledge, and in turn greater intentions to use and modify ENDS devices.

Moreover, in the United States, about 77% of individuals aged 18-25 years use YouTube [27]. This demographic group often experiments with cigarettes and ENDSs [28] and is frequently targeted by tobacco companies [29,30]. Indeed, when sharing their experiences of tobacco use and ENDS modifications, some viewers indicated that they started to use tobacco products at an early age (eg, “I used to smoke when I was 15,” and “At 16, my brother and I began to vape.”). It is alarming that modification videos, which may encourage ENDS use and modifications, are accessible to young audiences across the world. Thus, more attention should be devoted to the impacts of online modification information, such as YouTube videos, on people’s ENDS use and modifications.

In contrast to the positivity and support shown in most comments, only 5% (143/2859) of the comments directly mentioned the health risks and safety concerns of ENDS modifications and tobacco use. Moreover, among the 15 comments that explicitly mentioned regulations, 13 were against regulations of ENDS use and modifications. Only 2 comments clearly stated that certain ENDS products and modifications should be banned or regulated. The results are not surprising given that most modifications videos did not include a safety warning [13], and many viewers of the videos, especially those who left a comment, may have already had modification experiences as well as hold positive attitudes toward ENDS use and modifications. However, the small percentage of comments mentioning risks and regulations are indeed alarming. Tobacco regulatory sciences and agencies should be aware of the YouTube ENDS videos and investigate the cognitive, emotional, and behavioral effects of those modification videos on YouTube viewers.

### Limitations

There are several limitations of this study. First, we only collected comments directed to the videos and excluded replies to existing comments. While reply comments were infrequent in our data collection and our approach helped focus on how viewers respond to video content, we left the potential interactions and dynamics between viewers and creators for future research. Moreover, our decision to collect the top 20 comments of each video on one day may not capture the dynamics and full landscape of the comments. In addition, we focused on English comments only. Yet, many YouTube videos are accessible across the globe. We were unable to explore how non-English speaking viewers react to and think about ENDS use and modifications. Furthermore, we did not know the demographics and other characteristics of viewers who left comments. Moreover, although many comments mentioned that viewers had gained new knowledge and intended to modify their ENDS devices, no causal relationships can be established in a content analysis. Our results were descriptive in nature. Future experimental studies should explicitly investigate how modification videos affect viewers. Lastly, our sample was collected in early 2019, before the report of the first e-cigarette or vaping use-associated lung injury (EVALI) case and the COVID-19 pandemic. We do not know how those public health crises may affect ENDS modification activities.

### Conclusion

Notwithstanding the limitations, to the best of our knowledge, this is the first study exploring the content of YouTube ENDS modification video comments. Our results indicated the acceptance and popularity of ENDS modifications among users and potential users. Some comments also suggested that the videos motivated current and new ENDS users to alter their ENDS devices. Few comments mentioned the health risks and safety concerns of ENDS modification, and very few mentioned ENDS product and modification regulations, among which only 2 comments clearly supported regulations. Tobacco regulatory researchers and agencies should be aware of online ENDS modification information. More research and attention should be devoted to understanding the impacts of online modification messages.

## Abbreviations

CDC: The Centers for Disease Control and Prevention
EVALI: e-cigarette or vaping use-associated lung injury
